# Increased Plasma Levels of Myosin Heavy Chain 11 Is Associated with Atherosclerosis

**DOI:** 10.3390/jcm10143155

**Published:** 2021-07-16

**Authors:** Lisa Takahashi, Tomoaki Ishigami, Hirofumi Tomiyama, Yuko Kato, Hiroyuki Kikuchi, Koichiro Tasaki, Jun Yamashita, Shigeru Inoue, Masataka Taguri, Toshitaka Nagao, Taishiro Chikamori, Yoshihiro Ishikawa, Utako Yokoyama

**Affiliations:** 1 Department of Cardiology, Tokyo Medical University, 6-7-1 Nishi-shinjuku, Shinjuku-ku, Tokyo 160-0023, Japan; lisataka@tokyo-med.ac.jp (L.T.); tomiyama@tokyo-med.ac.jp (H.T.); jyamashi@tokyo-med.ac.jp (J.Y.); chikamd@tokyo-med.ac.jp (T.C.); 2 Department of Physiology, Tokyo Medical University, 6-6-1 Shinjuku, Shinjuku-ku, Tokyo 160-8402, Japan; yukato@tokyo-med.ac.jp; 3 Department of Cardio-Renal Medicine and Medical Science, Yokohama City University, 3-9 Fukuura, Yokohama 236-0004, Japan; tommmish@hotmail.com; 4 Department of Preventive Medicine and Public Health, Tokyo Medical University, 6-6-1 Shinjuku, Shinjuku-ku, Tokyo 160-8402, Japan; kikuchih@tokyo-med.ac.jp (H.K.); inoue@tokyo-med.ac.jp (S.I.); 5 Department of Pathology, Tokyo Medical University, 6-7-1 Nishi-shinjuku, Shinjuku-ku, Tokyo 160-0023, Japan; k-tasaki@tokyo-med.ac.jp (K.T.); nagao-t@tokyo-med.ac.jp (T.N.); 6 Department of Data Science, Yokohama City University, 22-2 Seto, Kanazawa-ku, Yokohama 236-0027, Japan; masataka.taguri@gmail.com; 7 Cardiovascular Research Institute, Yokohama City University, 3-9 Fukuura, Kanazawa-ku, Yokohama 236-0004, Japan; yishikaw@yokohama-cu.ac.jp

**Keywords:** atherosclerosis, immunohistochemistry, biomarkers, smooth muscle cells, myosin heavy chain

## Abstract

Many studies have revealed numerous potential biomarkers for atherosclerosis, but tissue-specific biomarkers are still needed. Recent lineage-tracing studies revealed that smooth muscle cells (SMCs) contribute substantially to plaque formation, and the loss of SMCs causes plaque vulnerability. We investigated the association of SMC-specific myosin heavy chain 11 (myosin-11) with atherosclerosis. Forty-five patients with atherosclerosis and 34 control subjects were recruited into our study. In the atherosclerosis patients, 35 patients had either coronary artery disease (CAD) or peripheral artery disease (PAD), and 10 had both CAD and PAD. Coronary arteries isolated from five patients were subjected to histological study. Circulating myosin-11 levels were higher in the CAD or PAD group than in controls. The area under the receiver operating characteristic curve of myosin-11 was 0.954. Circulating myosin-11 levels in the CAD and PAD group were higher than in the CAD or PAD group, while high-sensitivity C-reactive protein concentrations did not differ between these groups. Multinomial logistic regression analyses showed a significant association of myosin-11 levels with the presence of multiple atherosclerotic regions. Myosin-11 was expressed in the medial layer of human atherosclerotic lesions where apoptosis elevated. Circulating myosin-11 levels may be useful for detecting spatial expansion of atherosclerotic regions.

## 1. Introduction

Atherosclerosis is the main pathological process underlying myocardial infarction, heart failure, peripheral artery disease (PAD), stroke, and cerebral infarction, and it has been the leading cause of morbidity and mortality globally [1]. Atherosclerotic plaque formation develops over long periods with chronic inflammation based on complex processes, including the oxidation of accumulated cholesterol-carrying low-density lipoprotein (LDL), immune cell infiltration, production of inflammatory mediators, endothelial dysfunctions, and vascular smooth muscle cell (VSMC) proliferation and migration [2,3]. Plaque instability, in which the activation of proteases for extracellular matrices and the loss of VSMCs are involved, increases the risk of cardiovascular events [4]. Because these pathological changes in the vascular wall are mostly asymptomatic, the detection of atherosclerotic lesions by serological biomarkers before critical clinical features emerge has been extensively investigated [5,6,7,8,9].

In atherosclerotic plaques, differentiated VSMCs that are derived from the tunica media undergo phenotypic switching to proliferative synthetic cells that produce extracellular matrices (ECMs) and contribute to plaque stabilization [10]. In vulnerable plaques, previous studies indicated a high proportion of infiltrated monocytes/macrophages and extracellular lipids rather than VSMCs [4]. Thus, VSMCs have been thought to play relatively minor roles in the progression to rupture-prone atherosclerosis [1,4,10]. Over the past decade, however, studies that used fate-mapping and lineage-tracing revealed that VSMCs account for up to 70% of all plaque cells in murine models of atherosclerosis and that VSMCs contribute to multiple plaque cell phenotypes, i.e., macrophage-like cells, foam cells, osteochondrogenic cells, and mesenchymal stem cells alongside ECM-producing α-smooth muscle actin (αSMA)-positive cells [11,12,13,14,15], which highlight the importance of VSMCs. Thus, VSMCs are a major cell type in plaque formation and play a greater role in atherosclerosis than previously recognized [1].

Loss of VSMCs through cell death, including apoptosis and secondary necrosis, was shown to occur during the progression of atherosclerosis [16,17]. In studies using animal models, relatively acute VSMC apoptosis induced features of plaque vulnerability, such as fibrous cap thinning [18], and chronic apoptosis of VSMCs accelerated plaque growth necrotic core enlargement, plaque calcification, medial expansion and degeneration, elastin breaks, and failure of outward remodeling [19]. In humans, a decrease in the VSMC cell number was correlated with plaque instability [4]. On the basis of these findings, we hypothesized that SMC-specific proteins leak into the circulation from dying cells with the development and spread of atherosclerotic regions.

Some of myosin superfamily members were expressed in a cell-type-specific manner [20] and were reported to be increased in the blood of patients with several diseases, including autoimmune diseases [21], myocardial cell damages [22,23], and skeletal muscle injuries [24]. Myosin heavy chain 11 (myosin-11) is exclusively enriched in VSMCs [25]. A recent report demonstrated that patients with a non-ruptured abdominal aortic aneurysm (AAA), in which VSMCs undergo apoptosis, had significantly higher levels of circulating myosin-11 than normal controls, and its levels were correlated with the maximum aortic diameter [26]. These data indicated that circulating myosin-11 levels are associated with the loss of VSMCs in the vascular wall. We investigated the association of plasma levels of myosin-11 and atherosclerosis to identify a new tissue-specific serological biomarker for atherosclerosis.

## 2. Materials and Methods

### 2.1. Study Subjects

Forty-five patients with atherosclerosis were recruited from June in 2013 to January in 2015 at Yokohama City University Hospital. Atherosclerosis patients consisted of two groups: 35 patients with either coronary artery disease (CAD) or PAD (the CAD or PAD group) and 10 patients with both CAD and PAD (the CAD + PAD group). Plasma samples were taken within 1 week before the patients received percutaneous coronary artery intervention or endovascular treatment. Plasma samples from 34 control subjects were collected from age- and sex-ratio-matched healthy volunteers at Maebashi Hirosegawa Clinic who did not have a history of CAD, PAD, cerebrovascular disease, aortic aneurysm, diabetes mellitus, or renal insufficiency. Medical interviews were performed when the subjects were recruited into this study, and their systolic and diastolic blood pressure and body mass index (BMI) were recorded at that time. Plasma samples were stored at −80 °C until analysis. The proximal segments of the left coronary arteries from five patients (2–5 segments per patient) were collected at autopsies in 2020 at Tokyo Medical University Hospital. Tissues were fixed in 4% paraformaldehyde for histological analysis. 

### 2.2. Clinical and Biochemical Analysis

Plasma levels of myosin-11 were quantified in a specific sandwich enzyme-linked immunosorbent assay (ELISA) (Cusabio Biotech, College Park, MD, USA) in accordance with the manufacturer’s instructions. Although the information on antibody-binding sites was not disclosed by the company, the ELISA was reacted to human recombinant coiled-coil domain of myosin-11 (data not shown). High-sensitivity C-reactive protein (hsCRP) was measured by SRL, Inc. (Tokyo, Japan).

### 2.3. Tissue Staining and Immunohistochemistry

Paraffin-embedded blocks containing the coronary artery tissues were cut into 4 μm–thick sections. Elastica van Gieson staining and Masson’s trichrome staining were performed for morphological analysis. We used anti-CD68 (1:200 dilution, M0876, Dako, Carpinteria, CA, USA) to detect macrophages in immunohistochemical staining.

The 5′ and 3′ end of *Myh11* (myosin-11 gene) are alternatively spliced to generate COOH-terminal isoforms (SM1 and SM2) and NH_2_-terminal isoforms (SM-A and SM-B) [27]. Among all combinations of the four isoforms (SM1A, SM1B, SM2A, and SM2B) identified in humans [28], the artery expressed exclusively SM1A and SM2A [27]. We, therefore, used anti-SM1 (1:500 dilution, 7600, Yamasa, Chuo-ku, Tokyo, Japan), and anti-SM2 (1:200 dilution, 7601, Yamasa, Chuo-ku, Tokyo, Japan) antibodies to detect myosin-11 isoforms. Biotinylated horse anti-mouse IgG (Vectastain Elite ABC IgG kit, Vector Labs, Burlingame, CA, USA) was used as a secondary antibody. Negative staining of immunohistochemistry was confirmed by the omission of primary antibodies. TdT-mediated dUTP Nick End Labeling (TUNEL) (G7130, Promega Corporation, Madison, WI, USA) was performed to evaluate apoptotic cells.

### 2.4. Statistical Analysis

**Figure 1:**
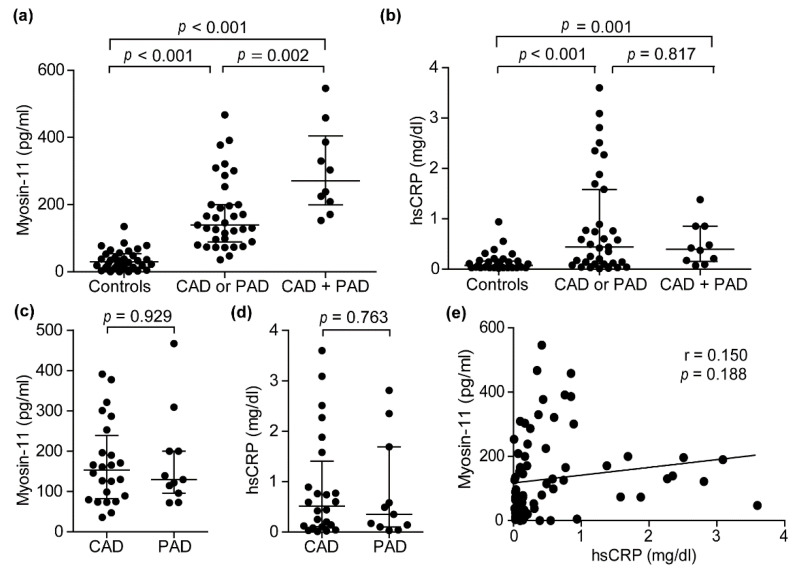
Plasma myosin-11 (myosin heavy chain 11) levels in patients with atherosclerosis. (**a**,**b**) Myosin-11 and hsCRP (high-sensitivity C-reactive protein) concentrations in plasma samples of control subjects (Controls, *n* = 34), patients with CAD (coronary artery disease) or PAD (peripheral artery disease) (CAD or PAD, *n* = 35), and patients with both CAD and PAD (CAD + PAD, *n* = 11). Data are shown as the median with interquartile ranges. (**c**,**d**) Myosin-11 and hsCRP concentrations in plasma samples from CAD (*n* = 24) and PAD (*n* = 11) patients. Data are shown as the median with interquartile ranges. (**e**) Correlation between plasma concentrations of myosin-11 and hsCRP in all patients and controls (*n* = 80).

**Figure 2:**
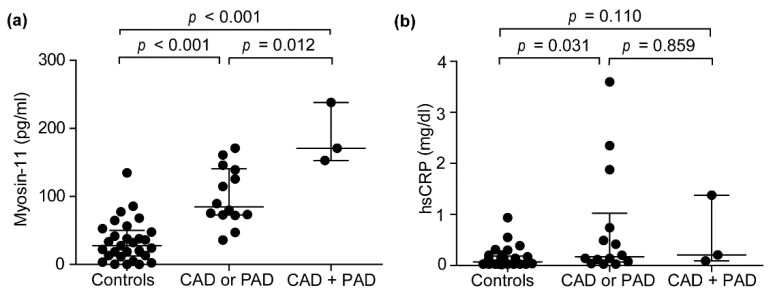
Plasma myosin-11 (myosin heavy chain 11) levels in subjects without renal insufficiency. (**a**,**b**) Myosin-11 and hsCRP (high-sensitivity C-reactive protein) concentrations in plasma samples of control subjects (Controls, *n* = 29), patients with CAD (coronary artery disease) or PAD (peripheral artery disease) (CAD or PAD, *n* = 14), and patients with both CAD and PAD (CAD + PAD, *n* = 3). Data are shown as the median with interquartile ranges.

In Table 1, the data that are shown as values were statistically analyzed by using the Kruskal-Wallis test, and the data shown as ratios were analyzed by using the chi-square test. Both of these tests were followed by Fisher’s least significant difference post hoc test, the Mann-Whitney U-test, or Fisher’s exact test, as appropriate. In Table 2, the data that are shown as values were statistically analyzed by using the Mann-Whitney U-test, and the data shown as ratios were analyzed by Fisher’s exact test. The data in Figure 1a,b and Figure 2a,b were statistically analyzed by using the Kruskal-Wallis test, followed by Fisher’s least-significant-difference post hoc test and the Mann-Whitney U-test. The Mann-Whitney U-test was used in Figure 1c,d. Spearman’s correlation analysis was used in Figure 1e. Data were analyzed by using Prism software (version 5.0; GraphPad, San Diego, CA, USA). Receiver-operating characteristic (ROC) analysis was performed by using a binary analysis of factors to evaluate the diagnostic performance. Multinomial logistic regression analyses were used to assess the difference of hsCRP (Model 1) and myosin-11 (Model 2) values within the three groups, after adjusting the Brinkman index (per 100), hypertension (dichotomous), and dyslipidemia status (dichotomous). Before the analysis, both myosin-11 and hsCRP values were transformed into the standardized score (z-score) for comparability. ROC analysis was examined by using SPSS (version 26; IBM Corp., Armonk, NY, USA), and multinomial logistic regression analyses were performed by using Stata (version 15; Stata Corp., College Station, TX, USA). The *p*-values < 0.05 were considered to be statistically significant.

## 3. Results

### 3.1. Characteristics of Patients with Atherosclerosis

Clinical baseline characteristics of patients with atherosclerosis (*n* = 45) and control subjects (*n* = 34) are shown in Table 1. Patients with atherosclerosis were divided into two groups: the CAD or PAD group (*n* = 35), in which patients had either CAD or PAD, and the CAD + PAD group (*n* = 10), in which patients had both CAD and PAD. There were no significant differences in age, frequencies of male gender, or BMI between the three groups. Both the CAD or PAD group and the CAD + PAD group had a higher frequency of hypertension, dyslipidemia, smokers, renal dysfunction, and diabetes mellitus than the control group, and there were no differences in the frequencies of these populations between the CAD or PAD and the CAD + PAD groups. Use of statins, angiotensin-converting enzyme (ACE) inhibitors, angiotensin receptor blockers (ARBs), β-blockers, and acetylsalicylic acid was also more common among patients with atherosclerosis compared to control subjects. With the exception of statins, there were no differences in the usage of these medications between the CAD or PAD and the CAD + PAD groups.

### 3.2. Myosin-11 Plasma Levels Were Upregulated in Patients with Atherosclerosis

We measured plasma myosin-11 concentrations in patients with atherosclerosis patients and control subjects using an ELISA that was specific for myosin-11. Plasma myosin-11 levels were higher in the CAD or PAD group (median (25th–75th percentiles): 139.2 (89.3–200.0) pg/mL) and the CAD + PAD group (median (25th–75th percentiles): 252.1 (208.6–386.3) pg/mL) than in control subjects (median (25th–75th percentiles): 30.0 (13.1–53.6) pg/mL) (Figure 1a). In addition, myosin-11 levels were significantly higher in the CAD + PAD group than in the CAD or PAD group (Figure 1a). In this sample set, we analyzed the plasma concentrations of hsCRP, which is the most extensively studied potential biomarker for atherosclerosis [29,30]. Moreover, hsCRP plasma levels were significantly higher in the CAD or PAD group (median (25th–75th percentiles): 0.44 (0.10–1.58) pg/mL) than in control subjects (median (25th–75th percentiles): 0.07 (0.03–0.16) pg/mL) (Figure 1b), while there was no difference in hsCRP between the CAD or PAD group and the CAD + PAD group (median (25th–75th percentiles): 0.42 (0.17–0.85) pg/mL) (Figure 1b).

### 3.3. Circulating Myosin-11 Levels in Patients with CAD or PAD

Next, we examined whether there was a difference in circulating myosin-11 levels between patients with CAD and PAD. The patient information is shown in Table 2. There were no differences in patient characteristics between CAD and PAD patients except for the frequencies of diabetes mellitus and usage of acetylsalicylic acid. Plasma levels of myosin-11 were similar between CAD patients (median (25th–75th percentiles: 153.5 (82.2–238.8 pg/mL) and PAD patients (median (25th–75th percentiles): 129.7 (96.1–200.0) pg/mL) (Figure 1c). Similar to myosin-11, there was no difference in hsCRP between CAD (median (25th–75th percentiles): 0.52 (0.11–1.41] pg/mL) and PAD (median (25th–75th percentiles): 0.35 (0.10–1.69) pg/mL) patients (Figure 1e). There was no positive association of plasma myosin-11 levels with hsCRP (Figure 1e).

### 3.4. Circulating Myosin-11 Levels and Clinical Parameters

We analyzed the values of myosin-11 or hsCRP together with traditional clinical risk factors. We performed multinomial logistic regression analyses by using hypertension, dyslipidemia, and the Brinkman index as risk factors. Because we recruited control subjects from among individuals who did not have a history of diabetes mellitus or renal insufficiencies, we did not include these factors in this analysis. The significant association between increased circulating myosin-11 levels and the presence of either CAD or PAD compared to control subjects persisted after adjustment for the risk factors (Table 3, Model 1). Similarly, hsCRP levels had a significant association with the presence of either CAD or PAD (Model 2). When we set the CAD or PAD group as a reference, the significant association between circulating myoin-11 levels and the presence of multiple regions of atherosclerosis (CAD and PAD) (Table 3, Model 1) remained after the adjustment. However, traditional risk factors, i.e., smoking history, hypertension, and dyslipidemia, and plasma levels of hsCRP were not associated with the presence of both CAD and PAD (Models 1 and 2).

### 3.5. The Effects of Renal Function on Circulating Myosin-11 Levels

Although the differences in creatinine levels and estimated glomerular filtration rate (eGFR) between the CAD or PAD group and the CAD + PAD group did not reach significance, atherosclerosis patients had a higher frequency of renal insufficiency. We subsequently investigated the plasma levels of myosin-11 in atherosclerosis patients and control subjects who did not have a renal insufficiency, which was defined by less than 60 mL/min/1.73 m^2^ of eGFR. In accordance with the results that are presented in Figure 1, plasma myosin-11 levels were significantly higher in the CAD or PAD group (median (25th–75th percentiles): 84.6 (72.77–140.9) pg/mL) and the CAD + PAD group (median (25th–75th percentiles): 170.9 (152.8–238.0) pg/mL) than in control subjects (median (25th–75th percentiles): 27.5 (12.3–50.1) pg/mL) (Figure 2a). Circulating myosin-11 levels were also higher in the CAD + PAD group than in the CAD or PAD group (Figure 2a). In this dataset, plasma levels of hsCRP were significantly higher in the CAD or PAD group (median (25th–75th percentiles): 0.17 (0.07–1.03) pg/mL) than in control subjects (median (25th–75th percentiles): 0.07 (0.03–0.19) pg/mL) (Figure 2b), while hsCRP levels did not differ between the CAD or PAD group and the CAD + PAD group (median (25th–75th percentiles): 0.21 (0.09–1.38) pg/mL) (Figure 2b).

### 3.6. Efficacy of Myosin-11 for Diagnosis

**Figure 3:**
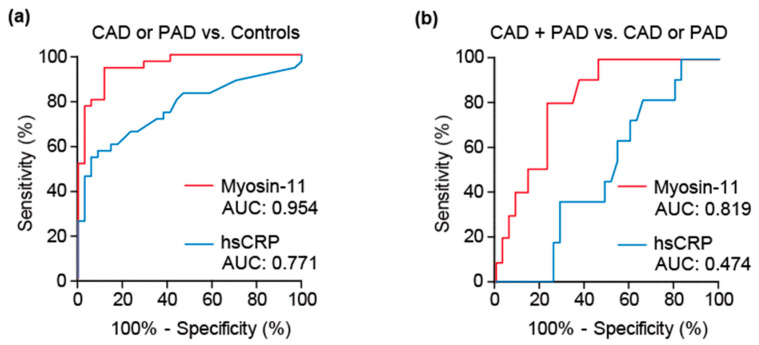
ROC (receiver-operating characteristic) analysis of myosin-11 (myosin heavy chain 11) and hsCRP (high-sensitivity C-reactive protein). (**a**) ROC curves of myosin-11 and hsCRP in patients with CAD (coronary artery disease) or PAD (peripheral artery disease) and control subjects. (**b**) Receiver-operating characteristic curves of myosin-11 and hsCRP in patients with CAD + PAD and CAD or PAD. AUC: the area under the curve.

We evaluated the diagnostic value using ROC analysis of myosin-11 to detect the presence of either CAD or PAD. The area under the curve (AUC) of myosin-11 was 0.954 (95% confidence interval [CI]: 0.909–0.998, *p* < 0.001), with a specificity of 88% at a sensitivity of 90% (Figure 3a), and the positive predictive value, negative predictive value, accuracy, and cutoff value were 89%, 91%, 90%, and 72.5 pg/mL, respectively. The AUC of hsCRP was 0.771 (95% CI: 0.657–0.884, *p* < 0.001), with a specificity of 29% at a sensitivity of 90%, and the positive predictive value, negative predictive value, and accuracy were 56%, 71%, and 59%, respectively. The AUC of myosin-11 was significantly greater than that of hsCRP (*p* < 0.025) (Figure 3a).

To further investigate the efficacy of myosin-11 to detect the presence of multiple regions of atherosclerosis, we compared circulating myosin-11 levels between the CAD or PAD group and the CAD + PAD group. The AUC of myosin-11 was 0.814 (95% CI: 0.691–0.938, *p* = 0.002), with a specificity of 63% at a sensitivity of 91% (Figure 3b), and the positive predictive value, negative predictive value, accuracy, and cutoff value were 43%, 96%, 70%, and 168.4 pg/mL, respectively. The AUC of hsCRP was 0.522 (95% CI: 0.339–0.705, *p* = 0.827), with a specificity of 20% at a sensitivity of 91%. The positive predictive value, negative predictive value, and accuracy were 26%, 88%, and 37%, respectively. The AUC of myosin-11 was significantly greater than that of hsCRP (*p* = 0.007) (Figure 3b). These results suggest that circulating myosin-11 levels were increased in patients with atherosclerosis, and its levels may reflect the spatial expansion of atherosclerotic regions.

### 3.7. Expression of Myosin-11 Isoforms in the Coronary Arteries

Finally, we investigated myosin-11 expression and apoptosis during atherosclerosis progression in humans. The patient information is shown in Table 4.

**Figure 4:**
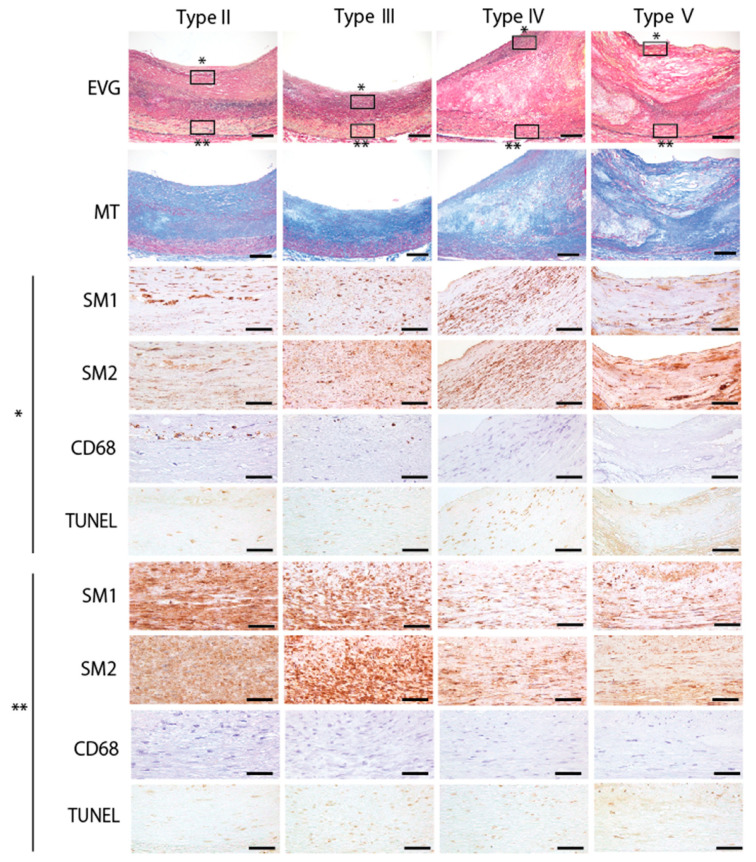
Histological analysis of human coronary arteries. Representative images of EVG (Elastica van Gieson staining), MT (Masson’s trichrome staining), and immunohistochemistry for CD68 (a marker of macrophages), SM1 and SM2 (myosin heavy chain 11 isoforms), and TUNEL (TdT-mediated dUTP Nick End Labeling) stain in Type II (patient 4), Type III (patient 1), Type IV (patient 3), and Type V (patient 3) atherosclerotic lesions. * Magnified images of black boxes in the intimal layer. ** Magnified images of black boxes in the medial layer. Scale bars for EVG and MT: 200 μm. Scale bars for immunohistochemistry and TUNEL stain: 50 μm.

Proximal regions in human left coronary arteries were evaluated based on the American Heart Association (AHA)-recommended classification of atherosclerotic lesions [31]. Expression of SM1 and SM2 was greatly decreased for both in the intimal layer in Type II through Type V lesions, except in the shoulder region of atheromatous plaque in Type IV lesion and in intraplaque neovasculatures in Type V lesion defined as those in which major parts of the fibromuscular cap represent replacement of tissue disrupted by accumulated lipid and hematoma or organized thrombotic deposits (Figure 4). These findings were consistent with a previous report [28]. Expression of both SM1 and SM2 was gradually decreased in the medial layer as atherosclerosis progressed, while both expression levels were relatively maintained in the medial layer compared to that in the intimal layer (Figure 4). 

TUNEL-positive apoptotic cells were present in the intimal layer in Type II lesion and more advanced atherosclerotic lesions where macrophages were accumulated, as reported previously [32]. In the medial layer, apoptosis was increased in Types III, IV, and V lesions, in which moderate expression of both SM1 and SM2 proteins was observed (Figure 4). These apoptotic cells seemed originate from SMCs, because CD68-positive cells were rarely seen in these areas. 

## 4. Discussion

During the past decade, the importance of VSMCs in atherosclerosis pathology has been re-evaluated [1]. Considerable efforts to identify subjects who are at a higher risk for cardiovascular events have been made, and numerous biomarkers were proposed [5,9]. The associated pathological processes are complex, and proteins related to lipid, inflammation, oxidative stress, coagulation, neurohumoral activity, and myocardial damage have been shown to be associated with the presence of atherosclerosis and vulnerability of atherosclerotic plaques [33]. Although recent studies identified micro-RNA enriched in VSMCs as a potential biomarker for atherosclerosis [5,34], vascular-tissue-specific circulating biomarkers are not currently available. In the present study, we focused on the VSMC-enriched protein myosin-11 and demonstrated increased plasma levels of myosin-11 in patients with CAD or PAD compared to control subjects. Plasma myosin-11 levels did not differ between patients with CAD and PAD, and circulating myosin-11 levels were higher in the CAD + PAD group than in the CAD or PAD group, suggesting that a higher level of circulating myosin-11 is associated with the presence of multiple atherosclerotic regions.

Pathological intimal thickening, which is the first stage of atherosclerosis, progresses to fibroatheromas, which are characterized by the formation of a fibrous cap and a necrotic core [1]. Differentiated mature contractile VSMCs express contractile genes, such as αSMA and myosin-11 [35], and VSMCs in the early stage fibrous cap, which protects against plaque rupture, also express αSMA and myosin-11 [36]. Recent lineage-tracing studies involving the use of *Myh11* (myosin-11 gene)-CreERT2 have demonstrated that VSMC-derived cells contributed substantially to the generation of a plaque core that is composed of αSMA-positive cells, macrophage-like cells, osteochondrogenic cells, and mesenchymal stem-cell-like cells [1,11,13,14,15]. Most αSMA-positive cells within the fibrous cap are positive for the VSMC-lineage label [37,38,39]. In the late stage of atherosclerosis, apoptosis is a hallmark of advanced atherosclerotic regions in humans [40]. It was reported that VSMC uptake of oxidized LDL and VSMC-derived foam cell formation seemed to induce VSMC apoptosis [17]. Induction of VSMC apoptosis using SM22α-hDTR/ApoE−/− mice induced fibrous cap thinning, necrotic core enlargement, plaque calcification, and medial degeneration, indicating that VSMC apoptosis accelerates atherosclerosis progression [19]. These data suggest that VSMCs proliferate during the early stage of atherosclerosis, and their loss in the arteries promotes plaque instability. Because myosin-11 is the component of smooth muscle myosin that is a major cytoskeletal/contractile protein of VSMCs and is theoretically not secreted from cells, increased circulating myosin-11 levels were thought to reflect dying VSMCs. In the present study, we measured plasma concentrations of myosin-11 in patients with advanced atherosclerosis in the coronary and peripheral arteries, so we do not know how early a stage of atherosclerosis we could possibly detect. The histology data in the present study demonstrated that expression the of both SM1 and SM2 was greatly decreased in the intimal layer, as reported previously [28], but were relatively maintained in the medial layer of advanced atherosclerotic lesions of human coronary arteries. Apoptosis in VSMCs seemed to be elevated in the medial layer of Type III lesions. Although the immunohistochemistry did not demonstrate that apoptotic SMCs in the medial layer are the source of circulating myosin-11, myosin-11 derived from VSMCs in Type III, as well as in Types IV and V, lesions may possibly affect circulating myosin-11 levels. Further study investigating the association of circulating myosin-11 levels with circulating apoptosis markers, such as cytokeratin-18 M30 antigen [41] and nucleosomes [42], at an asymptomatic early stage in atherosclerosis patients would clarify the timing at which circulating myosin-11 is elevated during atherosclerosis progression.

In a previous study, we conducted a secretome-based analysis of human AAA tissues and found that myosin-11 was abundantly detected in the supernatants of an organ culture of advanced AAA tissues [26]. In addition to the supernatants, circulating myosin-11 levels were increased in patients with AAA, and its levels were correlated with maximum aortic diameter [26]. It is widely recognized that aortic aneurysms that are localized at the abdominal region develop based on atherosclerotic pathological remodeling, and an advanced aneurysmal wall exhibits VSMC apoptosis and medial degeneration [43,44]. Another line of study also demonstrated that circulating myosin-11 levels were significantly increased in the blood immediately after aortic dissection occurred [45,46]. Together with the present study, these findings support the concept that elevated circulating myosin-11 levels seem to reflect the degree of VSMC loss.

An increasing amount of proteomic evidence has identified several circulating protein markers for atherosclerosis [9]. Although contractile genes are downregulated when VSMCs undergo phenotypic switching during atherosclerosis progression [47], VSMC-related cytoskeleton and contracting proteins were identified in tissue extracts and secretome analyses of human atherosclerotic samples, such as carotid or coronary plaques [9]. To the best of our knowledge, however, molecular signatures of circulating myosin-11 in blood derived from patients with atherosclerosis remain uncertain. 

VSMCs in the tunica media arise from local progenitor cells, and multiple distinct cell lineages are distributed across the arterial tree [48]. Coronary arteries are derived from pro-epicardium (lateral plate mesoderm) [49]. The infrarenal abdominal aorta and its distal peripheral arteries are derived from the splanchnic mesoderm [50]. A study using liquid chromatography/tandem mass spectrometric analysis (LC–MS/MS) analyses of the human AAA tunica media that is located in infra-renal regions demonstrated that the number of segments in a coiled-coil domain was larger than that of a motor domain in human AAA tissue secretion [26]. Because the infrarenal abdominal aorta and the peripheral arteries share a similar cell lineage, a coiled-coil domain of myosin-11 may be elevated in the blood of PAD patients. The present study, however, did not reveal molecular signatures of myosin-11 detected in the blood of CAD and PAD, because the ELISA used in this study could detect both fragments of myosin-11 containing a coiled-coil domain and full-length myosin-11. Identification of circulating myosin-11 by using mass spectrometric analysis and Western blotting in patients with atherosclerosis of each region, i.e., the coronary and peripheral arteries, would clarify whether myosin-11 in the blood is fragmented or full-length, and provide further information for atherosclerosis pathologies and developing a detection system.

There are several limitations to the present study. The present work was conducted by using a small number of samples, all with advanced atherosclerosis, and therefore, it lacks clinical background variety. Myosin-11 is abundantly expressed in arterial smooth muscle cells, but it is also found in the bladder, intestine, stomach, and uterus [27]. It is theoretically possible that circulating myosin-11 levels are elevated in patients with diseases of these organs. In addition to AAA and dissection of the aorta, circulating myosin-11 levels can be elevated in other vascular diseases, such as aortitis. The effect of gender on circulating myosin-11 levels remains unknown, because the number of female patients in this study was too small for statistical analysis.

The present study involved a higher frequency of renal insufficiency in atherosclerosis patients. A previous study reported that renal function did not increase circulating myosin-11 levels [46], and an analysis involving subjects without renal insufficiency in the present study demonstrated a similar tendency as that seen in the results using all subjects (Figure 2a,b). However, the effect of renal function on circulating myosin-11 levels should be considered, and the clearance of circulating myosin-11 needs to be examined in future studies. Because the progression of atherosclerosis consists of multiple pathological molecular processes, it is unlikely that a single molecule could be used to detect relatively early phase asymptomatic atherosclerosis and to monitor plaque vulnerability.

## 5. Conclusions

In conclusion, together with other biomarkers, circulating levels of myosin-11, which seem to reflect VSMC loss or damage, may help to detect the presence of atherosclerosis and spatial expansion in atherosclerosis regions. 

## Figures and Tables

**Table 1:** Clinical baseline characteristics of patients with atherosclerosis and controls.

Variables	A	B	C	*p*-value			
	Controls (*n* = 34)	CAD or PAD (*n* = 35)	CAD + PAD (*n* = 10)	A vs. B vs. C	A vs. B	A vs. C	B vs. C
Age, years	71.2 ± 3.7	69.9 ± 7.4	72 ± 8.5	0.348			
Male gender, *n* (%)	28 (82.4)	30 (85.7)	8 (80.0)	0.923			
Body mass index	23.3 ± 1.2	22.8 ± 3.2	21.7 ± 3.0	0.383			
Systolic BP, mmHg	132 ± 12	131 ± 19	138 ± 22	0.477			
Diastolic BP, mmHg	80 ± 8	66 ± 14	71 ± 13	<0.001 *	<0.001 *	0.047 *	0.262
HDL-cholesterol, mg/dL	58 ± 16	52 ± 14	44 ± 12	0.042 *	0.073	0.024 *	0.262
LDL-cholesterol, mg/dL	118 ± 19	83 ± 23	96 ± 27	<0.001 *	<0.001*	0.018 *	0.105
Triglyceride, mg/dL	101 ± 43	118 ± 67	125 ± 68	0.575			
HbA1c, %	5.5 ± 0.4	6.3 ± 0.9	6.0 ± 0.7	<0.001 *	<0.001 *	0.047 *	0.358
Creatinine, mg/dL	0.7 ± 0.2	3.8 ± 4.7	6.6 ± 5.8	<0.001 *	<0.001 *	<0.001 *	0.25
eGFR, mL/min/1.73 m^2^	78 ± 17	45 ± 32	30 ± 33	<0.001 *	<0.001*	<0.001 *	0.198
Hemodialysis, *n* (%)	0 (0)	11 (31.4)	6 (60.0)	<0.001 *	<0.001 *	<0.001 *	0.179
Hypertension, *n* (%)	15 (44.1)	28 (80.0)	9 (90.0)	0.001 *	0.002 *	0.013 *	0.661
Dyslipidemia, *n* (%)	14 (41.2)	28 (80.0)	6 (60.0)	0.004 *	0.001 *	0.472	0.228
Brinkman index	356 ± 423	710 ± 511	948 ± 681	0.006 *	0.008 *	0.014 *	0.286
Diabetes mellitus, *n* (%)	0 (0)	19 (54.3)	6 (60.0)	<0.001 *	<0.001 *	<0.001 *	>0.999
Statins, *n* (%)	1 (2.9)	28 (80.0)	3 (30.0)	<0.001 *	<0.001 *	0.032 *	0.005 *
ACE inhibitor or ARB, *n* (%)	4 (11.8)	20 (57.1)	7 (70.0)	<0.001 *	<0.001 *	0.001*	0.716
β-blocker, *n* (%)	2 (5.9)	21 (60.0)	3 (30.0)	<0.001 *	<0.001 *	0.069	0.151
Acetylsalicylic acid, *n* (%)	0 (0)	30 (85.7)	9 (90.0)	<0.001 *	<0.001 *	<0.001 *	>0.999

Continuous variables are shown as the mean ± SD and categorical variables are expressed as the number (%). * *p* < 0.05; n, number of subjects; CAD, coronary artery disease; PAD, peripheral artery disease; BP, blood pressure; HDL, high-density lipoprotein; LDL, low-density lipoprotein; Hb, hemoglobin; eGFR, estimated glomerular filtration rate; ACE, angiotensin-converting enzyme; ARB, angiotensin receptor blocker; SD, standard deviation.

**Table 2:** Clinical baseline characteristics of patients with CAD or PAD.

Variables	A	B	*p*-Value
	CAD (*n* = 24)	PAD (*n* = 11)	A vs. B
Age, years	70 ± 8.2	70 ± 5.3	0.587
Male gender, *n* (%)	21 (87.5)	9 (81.8)	0.656
Body mass index	22.4 ± 2.9	23.8 ± 3.6	0.238
Systolic BP, mmHg	127 ± 19	139 ± 17	0.085
Diastolic BP, mmHg	65 ± 12	67 ± 18	0.687
HDL-cholesterol, mg/dL	50 ± 12	56 ± 17	0.494
LDL-cholesterol, mg/dL	86 ± 22	78 ± 26	0.163
Triglyceride, mg/dL	113 ± 67	126 ± 69	0.472
HbA1c, %	6.3 ± 0.9	6.5 ± 0.8	0.18
Creatinine, mg/dL	2.7 ± 4.0	6.1 ± 5.7	0.252
eGFR, mL/min/1.73 m^2^	49 ± 28	37 ± 39	0.43
Hemodialysis, *n* (%)	6 (25.0)	5 (45.5)	0.115
Hypertension, *n* (%)	19 (79.2)	9 (81.8)	0.856
Dyslipidemia, *n* (%)	20 (83.3)	8 (72.7)	0.466
Brinkman index	724 ± 561	679 ± 402	0.847
Diabetes mellitus, *n* (%)	10 (41.7)	9 (81.8)	0.027 *
Statins, *n* (%)	20 (83.3)	8 (72.7)	0.466
ACE inhibitor or ARB, *n* (%)	16 (66.7)	4 (36.4)	0.093
β-blocker, *n* (%)	16 (66.7)	5 (45.5)	0.234
Acetylsalicylic acid, *n* (%)	24 (100)	6 (54.5)	<0.001 *

Continuous variables are shown as the mean ± SD, and categorical variables are expressed as the number (%). * *p* < 0.05; *n*, number of subjects; CAD, coronary artery disease; PAD, peripheral artery disease; BP, blood pressure; HDL, high-density lipoprotein; LDL, low-density lipoprotein; Hb, hemoglobin; eGFR, estimated glomerular filtration rate; ACE, angiotensin-converting enzyme; ARB, angiotensin receptor blocker; SD, standard deviation.

**Table 3:** Association between standardized score of myosin-11/hsCRP and atherosclerosis.

		Controls		CAD or PAD		CAD + PAD	
	AOR	95%CI	*p*-Value	(Reference)	AOR	95%CI	*p*-Value
*Model 1*							
Myosin-11^1^	0.01	0.01–0.03	0.002 *	(1.00)	4.10	1.43–11.69	0.008 *
Brinkman index ^2^	0.71	0.49–1.03	0.065	(1.00)	1.07	0.92–1.24	0.070
Hypertension	0.03	0.01–0.90	0.044 *	(1.00)	8.76	0.24–323.81	0.598
Dyslipidemia	0.12	0.01–3.52	0.219	(1.00)	0.31	0.05–1.91	0.276
*Model 2*							
hsCRP ^1^	0.02	0.01–0.37	0.008 *	(1.00)	0.58	0.23–1.42	0.232
Brinkman index ^2^	0.92	0.80–1.05	0.208	(1.00)	1.10	0.96–1.26	0.183
Hypertension	0.20	0.05–0.85	0.029 *	(1.00)	2.12	0.21–21.37	0.523
Dyslipidemia	0.17	0.04–0.72	0.016 *	(1.00)	0.29	0.05–1.49	0.136

* *p* < 0.05. ^1^ Values were transformed into a standardized score (z-score) before this analysis. ^2^ The AORs (adjusted odds ratio) were calculated using a per-100 change to the index. Myosin-11, myosin heavy chain 11; hsCRP, high-sensitivity C-reactive protein; CAD, coronary artery disease; PAD, peripheral artery disease; AOR, adjusted odds ratio; 95%CI, 95% confidence interval.

**Table 4:** Patient characteristics for histological analyses.

	Age	Gender	Diagnosis	Hypertension	Dyslipidemia	Diabetes Mellitus	Smoking History	Atherosclerosis Lesions
Patient 1	48	M	HCC	−	−	−	−	Type II, III
Patient 2	66	M	Liver cirrhosis	−	−	−	+	Type II, III
Patient 3	71	M	Lung cancer	+	−	+	+	Type III, IV, V
Patient 4	72	M	HCC	−	−	−	+	Type II, III
Patient 5	88	F	CHF	+	−	−	+	Type III, IV, V

HCC, hepatocellular carcinoma; CHF, chronic heart failure.

## Data Availability

The data presented in this study are available on request from the corresponding author. The data are not publicly available due to privacy.
